# Determinants of longitudinal change in insulin clearance: the Prospective Metabolism and Islet Cell Evaluation cohort

**DOI:** 10.1136/bmjdrc-2019-000825

**Published:** 2019-11-24

**Authors:** Zhila Semnani-Azad, Luke W Johnston, Christine Lee, Ravi Retnakaran, Philip W Connelly, Stewart B Harris, Bernard Zinman, Anthony J Hanley

**Affiliations:** 1 Department of Nutritional Sciences, University of Toronto, Toronto, Ontario, Canada; 2 Department of Public Health, Aarhus Universitet, Aarhus, Denmark; 3 Division of Endocrinology and Metabolism, University of Toronto, Toronto, Ontario, Canada; 4 Lunenfeld-Tanenbaum Research Institute, Mount Sinai Hospital, Toronto, Ontario, Canada; 5 Leadership Sinai Centre for Diabetes, Mount Sinai Hospital, Toronto, Ontario, Canada; 6 Keenan Research Centre for Biomedical Science, St Michael’s Hospital, Toronto, Ontario, Canada; 7 Centre for Studies in Family Medicine, Western University, London, Ontario, Canada

**Keywords:** clearance and action, beta cell function, insulin sensitivity, type 2 diabetes

## Abstract

**Objective:**

To evaluate multiple determinants of the longitudinal change in insulin clearance (IC) in subjects at high risk for type 2 diabetes (T2D).

**Research design and methods:**

Adults (n=492) at risk for T2D in the Prospective Metabolism and Islet Cell Evaluation cohort, a longitudinal observational cohort, had four visits over 9 years. Values from oral glucose tolerance tests collected at each assessment were used to calculate the ratios of both fasting C peptide-to-insulin (IC_FASTING_) and areas under the curve of C peptide-to-insulin (IC_AUC_). Generalized estimating equations (GEE) evaluated multiple determinants of longitudinal changes in IC.

**Results:**

IC declined by 20% over the 9-year follow-up period (p<0.05). Primary GEE results indicated that non-European ethnicity, as well as increases in baseline measures of waist circumference, white cell count, and alanine aminotransferase, was associated with declines in IC_FASTING_ and IC_AUC_ over time (all p<0.05). There were no significant associations of IC with sex, age, physical activity, smoking, or family history of T2D. Both baseline and longitudinal IC were associated with incident dysglycemia.

**Conclusions:**

Our findings suggest that non-European ethnicity and components of the metabolic syndrome, including central obesity, non-alcoholic fatty liver disease, and subclinical inflammation, may be related to longitudinal declines in IC.

**Significance of this study**

**What is already known about this subject?**

- Insulin clearance is an important regulator of circulating insulin concentrations and a component of the pathophysiology of type 2 diabetes. Previous studies, mainly cross-sectional in design, have reported lower insulin clearance in the setting of obesity, type 2 diabetes and related complications.

**What are the new findings?**

- Non-European ethnicity and core components of metabolic syndrome—specifically markers of fatty liver and subclinical inflammation—impact longitudinal declines in insulin clearance.

**How might these results change the focus of research or clinical practice?**

- Our findings indicate the complexity of factors that impact insulin clearance change, highlighting an interplay of both upstream metabolic abnormalities and compensatory responses to early metabolic disorders related to type 2 diabetes.

## Introduction

Impaired insulin sensitivity and suboptimal beta-cell function have been well documented as central pathophysiological disorders underlying type 2 diabetes (T2D).^1^ Although less extensively studied, insulin clearance (IC) is another important regulator of circulating plasma insulin concentrations, and previous studies have documented that reductions in IC are associated with the incidence of T2D and related to a number of its underlying abnormalities.^2–4^

The majority of circulating insulin is cleared by the liver through a receptor-substrate mechanism where it is degraded by the insulin-degrading enzyme (IDE), although smaller amounts of insulin are also cleared by the kidney and muscle.^5^ While factors that regulate IC have not been fully elucidated, IC has been reported to decline in the setting of obesity, T2D and related complications.^6–8^ It is hypothesized that declining IC may be a compensatory response to impaired insulin sensitivity and/or secretion, with a reduction in clearance contributing to the maintenance of adequate circulating insulin concentrations.^9 10^ In contrast, Kotronen and others have suggested that declining IC may in fact be a consequence of early dysmetabolic events, such as fatty liver.^8 11^

Exploring potential determinants of IC change can shed light on the role of IC in the development of downstream disorders such as T2D. Previous research has reported that IC is associated with cardiometabolic abnormalities. Two studies showed that components of the metabolic syndrome measured at baseline, including triglyceride, blood pressure and waist circumference (WC), were inversely associated with IC.^12 13^ Moreover, subjects with metabolic syndrome had lower IC compared with those without metabolic syndrome.^13^ Further, IC was inversely associated with alanine aminotransferase (ALT), a biomarker of fatty liver, as shown in cross-sectional^8 14 15^ and prospective cohort^2^ studies, while physical activity has been associated with increased IC.^16^

Although previous research has investigated the associations of cardiometabolic variables with IC, most studies have been cross-sectional or have compared baseline measures with IC change. Much less is known regarding the longitudinal relationships between potential determinants and changes in IC—particularly with the use of measurements conducted at multiple time points. Therefore, our objective was to assess baseline and longitudinal associations of a range of metabolic parameters with 9-year changes in IC using data from the Prospective Metabolism and Islet Cell Evaluation (PROMISE) cohort.

## Methodology

### Study population

The present study used data from the PROMISE cohort, a longitudinal observational cohort of adults aged >30 years with >1 risk factor for T2D including obesity, hypertension, a family history of diabetes, and/or history of gestational diabetes or birth of a macrosomic infant. Eligible consenting participants were recruited from London and Toronto, Canada, between 2004 and 2006 and follow-up visits occurred every 3 years (n*=*712).^17^ Participants were contacted annually by telephone. At each clinic visit, participants completed standard health and lifestyle questionnaires, and underwent anthropometric measurements and metabolic characterization. After excluding participants with baseline diabetes (n*=*54), and those with missing baseline IC measures (n*=*87) or missing follow-up IC measures (n*=*79), the remaining 492 participants were included in the current analysis (online supplementary figure 1).

10.1136/bmjdrc-2019-000825.supp1 Supplementary data

### Clinical measurements and procedures

#### Oral glucose tolerance test

Prior to each clinic visit, participants were asked to avoid smoking and strenuous exercise for 24 hours and fast for 8–12 hours preceding their oral glucose tolerance test (OGTT). Blood samples were collected after the overnight fast. A 75 g OGTT was administered after the fasting blood sample, with additional blood samples drawn at 30 and 120 min. Blood samples were processed, aliquoted and frozen at −80°C. C peptide, insulin and glucose concentrations at 0, 30, and 120 min of the OGTT were determined at the Banting and Best Diabetes Centre Core Lab at Mount Sinai Hospital (Toronto, Canada). C peptide was measured using Elecsys 2010 Cobas e411 immunoassay analyzer. Insulin was assessed using Elecsys 1010 immunoassay analyzer (Roche Diagnostics, Basel, Switzerland) and electrochemiluminescence immunoassays. Glucose was determined using an enzymatic hexokinase method (Roche Modular, Roche Diagnostics).

#### Anthropometric measurements and blood pressure

Blood pressure and anthropometric measurements were assessed twice and averaged. Blood pressure was measured using an automated sphygmomanometer on the right arm with the subject seated after resting for 5 min. WC and hip circumference were measured using standard procedures and used to calculate waist-to-hip ratio (waist:hip).

#### Standardized lifestyle questionnaires

Sociodemographic and lifestyle risk factors were assessed using structured, standardized questionnaires. Physical activity was determined using a version of the validated Modifiable Activity Questionnaire (MAQ).^18^ Using this questionnaire, information regarding leisure and occupational activity over the past year was determined. Each reported activity from the MAQ was weighted by its metabolic intensity allowing for the estimation of metabolic equivalent task (MET).

#### Biological markers

ALT and white cell count (WCC) were measured using standard laboratory procedures. Serum creatinine was used to calculate the estimated glomerular filtration rate (eGFR) as a measure of kidney function, using the Chronic Kidney Disease Epidemiology Collaboration equations.^19^

#### IC, insulin sensitivity, beta-cell function, pre-diabetes and dysglycemia

IC, insulin sensitivity, beta-cell function, pre-diabetes and dysglycemia were assessed using C peptide, insulin and/or glucose measures from OGTT.

IC was calculated using C peptide-to-insulin ratios, based on the assumption that C peptide and insulin are secreted in equimolar amounts but C peptide does not undergo hepatic first-pass metabolism.^20^ These ratios have been used in several published studies^13 21 22^ and have been validated against more detailed measures of IC determined in clamp experiments (IC_AUC_ r=0.74, p<0.001):

1.$IC_{FASTING}=\frac{FastingC-peptide}{FastingInsulin}$

2.$IC_{AUC}=\frac{C-peptideArea-Under-The-Curve\left(0-120mins\right)}{InsulinArea-Under-The-Curve\left(0-120mins\right)}$

Hepatic insulin sensitivity was determined by calculating the homeostasis model assessment (HOMA2) estimate of insulin sensitivity (HOMA2-%S)^23^ using fasting glucose and insulin data. Whole body insulin sensitivity was evaluated using the insulin sensitivity index (ISI).^24^ ISI takes into account fasting and mean insulin and glucose measures and has been validated against the hyperinsulinemic-euglycemic clamp technique (r=0.81).^24^ Beta-cell function was defined using the insulinogenic index (IGI) over HOMA of insulin resistance—a common method used in large studies as a measure of first-phase insulin secretion. IGI considers both fasting and 30 min insulin and glucose measures and has been validated against gold standard measures of insulin secretion.^25^ Beta-cell function was also defined using the insulin secretion-sensitivity index-2, a measure similar to the disposition index but calculated using OGTT data.^26^Online supplementary table 1 outlines the formulae used to calculate insulin sensitivity and beta-cell function.

Pre-diabetes was defined as impaired fasting glucose (IFG) and/or impaired glucose tolerance (IGT). Dysglycemia was defined as T2D, IFG or IGT. These definitions were based on OGTT measures according to 2006 WHO guidelines.^27^ Specifically, IFG was defined by fasting blood glucose measures between 6.1 and 6.9 mmol/L and IGT as fasting glucose <7.0 mmol/L and 2-hour OGTT blood glucose **≥**7.8 but <11.1 mmol/L. T2D was classified based on physician diagnosis, use of diabetes medication, or on fasting plasma glucose (FPG) level of ≥7.0 mmol/L or a 2-hour plasma glucose level ≥11.1 mmol/L^27^ during the OGTT.

### Statistical analyses

Baseline descriptive characteristics were presented across tertiles of IC_AUC_. Continuous variables were described as mean±SD and median with IQR for normally and non-normally distributed variables, respectively. Categorical variables were presented as number and percent. P values for continuous variables were determined by one-way analysis of variance and Kruskal-Wallis tests for normally and non-normally distributed variables, respectively. P values for categorical variables were determined using χ^2^ tests and Fisher tests. Baseline univariate associations of measures of IC with metabolic parameters were assessed through Spearman correlations.

Data from four time points (baseline, 3-year, 6-year, and 9-year visits) were used to assess longitudinal changes in IC and associations with potential determinants. For the primary analysis, generalized estimating equation (GEE) models^28^ were constructed to evaluate the associations of baseline measures of determinants with longitudinal change in our IC measures. GEE is an extension of the generalized linear model and provides a population estimate via a semiparametric approach to longitudinal analysis. It is used under the assumption that measurements within subjects are correlated. In addition, GEE accommodates missing values and allows for data from all visits to be included in the same model, maximizing statistical power.

The independent variables in the primary analysis were selected based on previous literature and biological plausibility, and included follow-up visit, sex, ethnicity, family history of T2D, age at baseline, WC, physical activity, smoking, WCC (marker of subclinical inflammation), ALT (marker of non-alcoholic fatty liver disease) and glycemic status. Values for predictors with only baseline measures were carried over for all four time points. GEE estimates were based on fully adjusted models for each predictor variable. All comparisons were further adjusted for eGFR to account for variation in renal C peptide clearance. Continuous predictor variables were scaled (mean centered and standardized) and the outcome variables, IC_AUC_ and IC_FASTING_, were both log transformed to achieve normality. Additionally, we examined determinants-by-time interactions for the two IC outcome variables.

GEE modeling was also conducted to determine the associations of baseline and longitudinal log-transformed measures of IC with dysglycemia. Models were adjusted for covariates including follow-up visit, sex, baseline age, ethnicity, physical activity, smoking status, family history of T2D, WCC, ALT, and eGFR. Continuous covariates were scaled. Results were presented as ORs using the ‘logit’ link function.

All analyses were performed using R V.3.4.3. GEE models were conducted using the R geepack package (https://cran.r-project.org/web/packages/geepack/index.html). For each GEE model, autoregressive of order 1 correlation matrix was selected. Benjamini-Hochberg false discovery rate correction was applied to account for multiple comparisons. Statistical significance was set at p<0.05.

## Results

Table 1 presents baseline demographic characteristics of participants by IC_AUC_ tertile distribution. Measures for IC_FASTING_ increased significantly with increasing IC_AUC_ (p<0.001). Age and physical activity increased across tertiles (both p<0.01). A higher proportion of those of European ethnicity were classified in the highest IC_AUC_ tertile; whereas the majority of participants classified as non-European were in the lowest IC_AUC_ tertile. There was an inverse association between obesity indicators (body mass index (BMI), BMI category, WC, and waist:hip) with IC_AUC_. In addition, eGFR, WCC and ALT significantly declined across increasing IC_AUC_ tertiles. There were no significant associations across IC_AUC_ tertiles for sex, smoking status, fasting glucose concentration, or glucose tolerance status.

**Table 1:** Baseline demographic, anthropometric and metabolic characteristics of PROMISE participants according to tertiles of IC_AUC_ (n=492)

	IC_AUC_ tertiles			P value
	1 (Lowest) [1.76–5.47]	2 [5.47–7.18]	3 (Highest) [7.18–18.29]	
Subjects, n	164	164	164	
Age (years)	47.84 (10.04)	50.13 (9.59)	51.80 (9.61)	0.001
Sex=male (%)	49 (29.9)	43 (26.2)	41 (25.0)	0.585
Ethnicity, n (%)				<0.001
European	96 (59.1)	116 (70.7)	135 (82.3)	
Other	67 (40.9)	48 (29.3)	29 (17.7)	
Smoking status, n (%)				0.392
Current	12 (7.5)	6 (3.8)	12 (7.4)	
Former	68 (42.8)	62 (38.8)	60 (36.8)	
Never	79 (49.7)	92 (57.5)	91 (55.8)	
Physical activity (kcal/kg/week)	15.45 [5.56, 40.86]	22.96 [8.49, 73.47]	27.40 [11.60, 69.24]	0.007
BMI (kg/m^2^)	32.54 (5.78)	31.23 (6.59)	29.22 (6.30)	<0.001
BMI category, n (%)				<0.001
Normal	9 (5.6)	24 (15.0)	40 (24.4)	
Overweight	49 (30.6)	50 (31.2)	62 (37.8)	
Obese	102 (63.7)	86 (53.8)	62 (37.8)	
Waist circumference (cm)	102.67 (14.74)	98.39 (14.24)	93.47 (15.82)	<0.001
Waist:hip	0.93 (0.07)	0.91 (0.07)	0.90 (0.08)	<0.001
IC_FASTING_	10.03 [8.49, 11.78]	12.87 [11.32, 15.46]	16.78 [13.88, 20.77]	<0.001
IC_AUC_	4.60 [3.97, 4.98]	6.28 [5.81, 6.67]	8.43 [7.77, 9.89]	<0.001
Fasting C peptide (pmol/L)	1021.50 [786.00, 1303.25]	805.50 [633.75, 1006.25]	661.50 [534.75, 863.25]	<0.001
Fasting glucose (mmol/L)	5.00 [4.60, 5.20]	4.80 [4.60, 5.20]	4.80 [4.50, 5.30]	0.091
Fasting insulin (pmol/L)	108.50 [77.75, 137.25]	60.00 [45.75, 80.00]	38.00 [27.00, 53.00]	<0.001
2-hour C peptide (pmol/L)	3340.00 [2515.00, 4264.50]	2692.00 [2058.75, 3353.25]	2256.50 [1850.75, 2960.75]	<0.001
2-hour glucose (mmol/L)	6.07 (1.33)	5.58 (1.22)	5.39 (1.50)	<0.001
2-hour insulin (pmol/L)	597.50 [418.50, 896.25]	322.00 [231.00, 460.25]	187.50 [135.00, 285.75]	<0.001
eGFR (mL/min/1.73 m^2^)	91.11 (18.35)	87.12 (16.68)	86.43 (17.61)	0.036
WCC (×10^9^/L)	6.29 (1.46)	6.00 (1.58)	5.60 (1.38)	<0.001
ALT (U/L)	29.50 [22.00, 39.75]	27.00 [20.50, 33.00]	25.00 [19.00, 31.00]	<0.001
Glucose tolerance status, n (%)				0.174
NGT	152 (92.5)	159 (96.9)	157 (95.6)	
Pre-diabetes	12 (7.5)	5 (3.1)	7 (4.4)	

Continuous values indicated as median [IQR] for non-normally distributed variables, otherwise presented as mean±SD. Categorical variables presented as number (percent). P values for continuous variables were determined by one-way analysis of variance (ANOVA) and Kruskal-Wallis tests for normally and non-normally distributed variables, respectively. P values for categorical variables were determined using χ^2^ tests and Fisher tests.

ALT, alanine aminotransferase; AUC, area under the curve; BMI, body mass index; eGFR, estimated glomerular filtration rate; IC, insulin clearance; NGT, normal glucose tolerance; PROMISE, Prospective Metabolism and Islet Cell Evaluation; WCC, white cell count.

Univariate associations between baseline IC measures and metabolic parameters are presented in table 2. IC_FASTING_ and IC_AUC_ were strongly correlated (r=0.71, p<0.05). Age was positively associated with both IC_FASTING_ and IC_AUC_ (r=0.19 and 0.17, both p<0.05, respectively). All measures of obesity (BMI, WC, and waist:hip) were inversely associated with IC_FASTING_ (r=−0.39 to −0.21) and IC_AUC_ (r=−0.29 to −0.20), all p<0.05. WCC, ALT and eGFR were inversely correlated with both IC measures (IC_FASTING_ r=−0.26 to −0.12 and IC_AUC_ r=−0.25 to −0.09, all p<0.05).

**Table 2:** Spearman correlations of metabolic parameters with IC_FASTING_ and IC_AUC_ at baseline

Parameter	IC_FASTING_	IC_AUC_
IC_AUC_	0.71*	
Age	0.19*	0.17*
BMI	−0.39*	−0.29*
Waist circumference	−0.35*	−0.27*
Waist:hip	−0.21*	−0.20*
WCC	−0.25*	−0.25*
ALT	−0.26*	−0.21*
eGFR	−0.12*	−0.09*

*P<0.05.

ALT, alanine aminotransferase; AUC, area under the curve; BMI, body mass index; eGFR, estimated glomerular filtration rate; IC, insulin clearance; WCC, white cell count.

**Figure 1:**
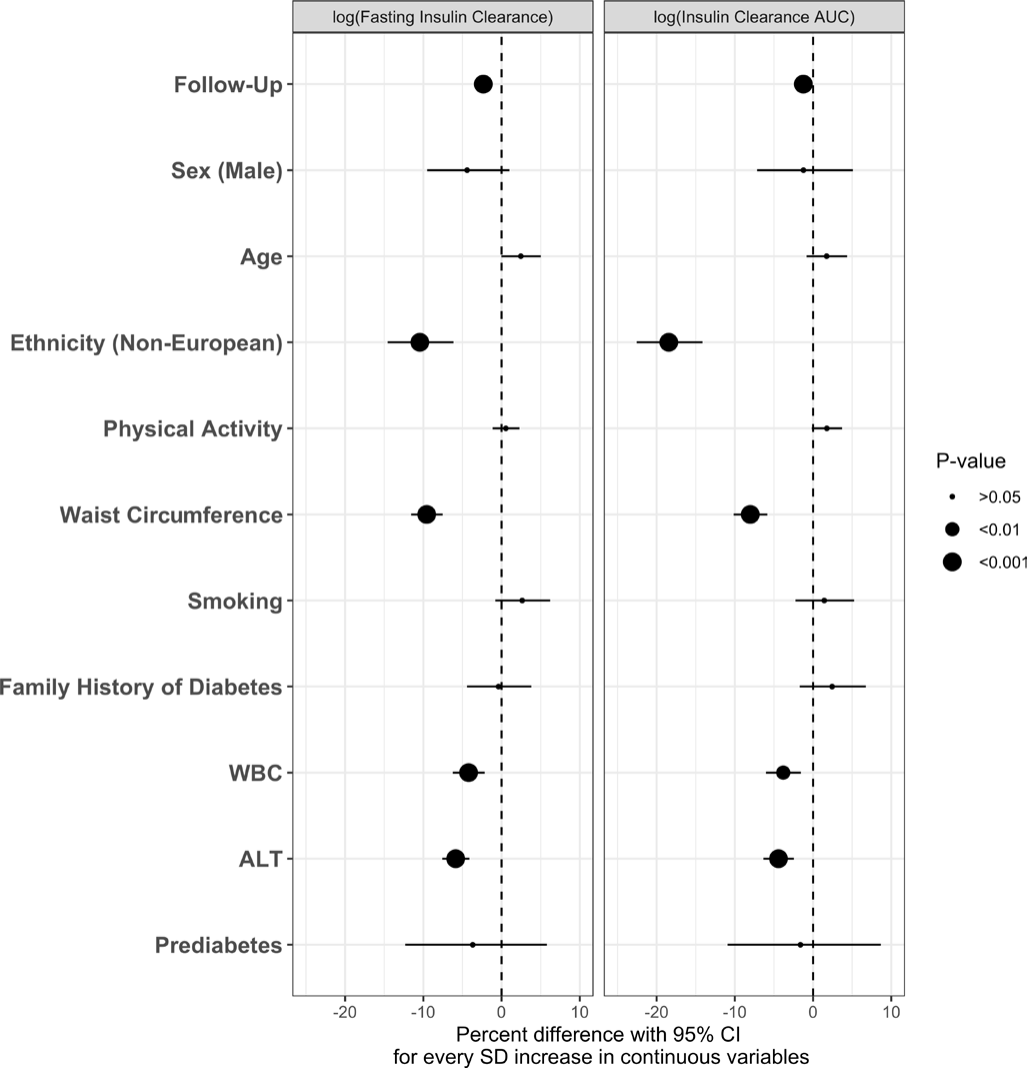
Generalized estimating equation (GEE) models showing the associations of individual baseline parameters with longitudinal IC_FASTING_ and IC_AUC_ over 9-year follow-up in the Prospective Metabolism and Islet Cell Evaluation (PROMISE) cohort. Models were fully adjusted. X-axis values represent percent difference (with 95% CI) in IC per SD increase for continuous determinants. P values were adjusted for the Benjamini-Hochberg false discovery rate. GEE model is adjusted for estimated glomerular filtration rate (eGFR). ALT, alanine aminotransferase; AUC, area under the curve; IC, insulin clearance; WBC, white blood cell count.

**Figure 2:**
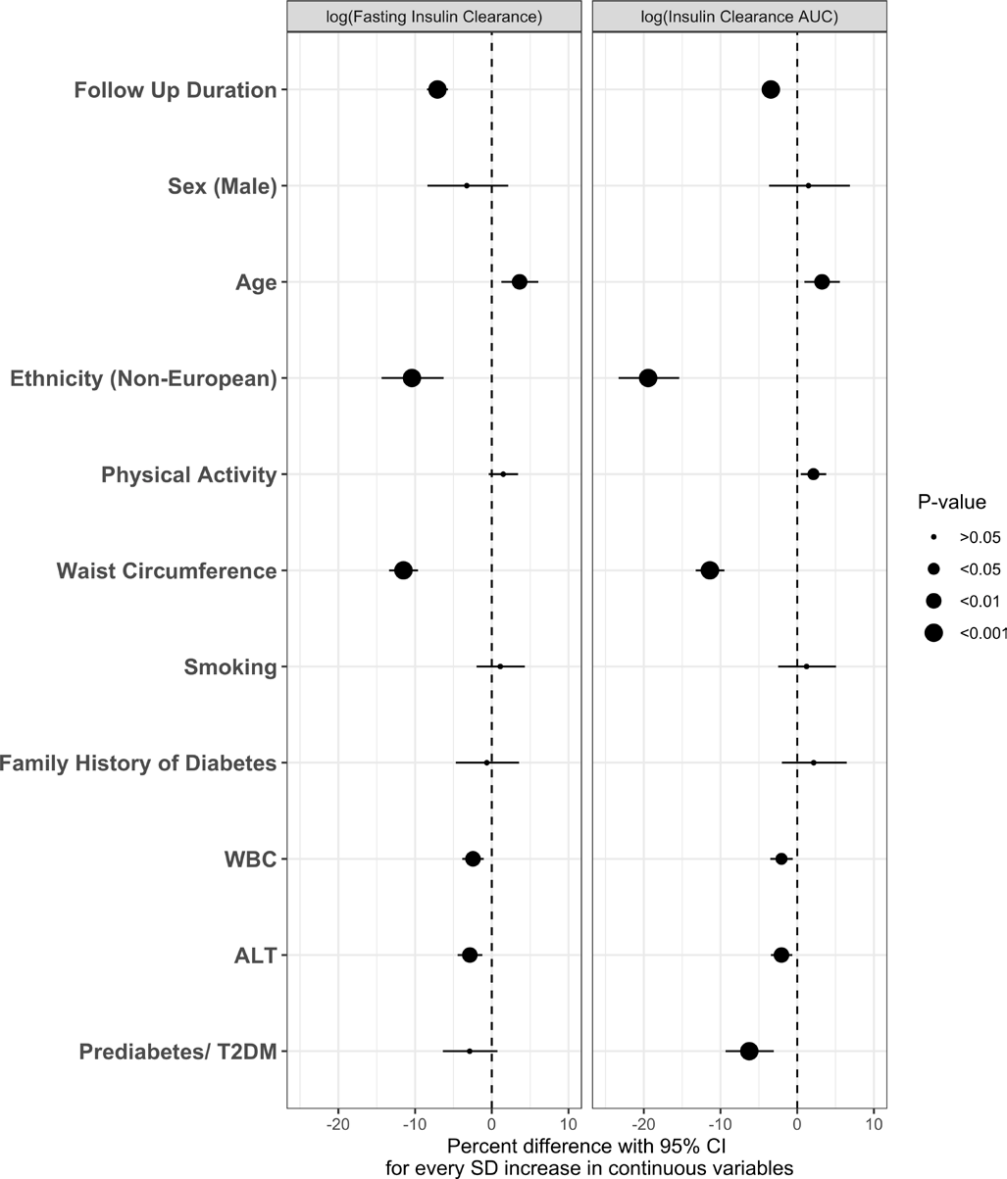
Generalized estimating equation (GEE) models showing the associations of individual longitudinal determinants with longitudinal IC_FASTING_ and IC_AUC_ over 9-year follow-up in the Prospective Metabolism and Islet Cell Evaluation (PROMISE) cohort. Models were fully adjusted. Variables only measured at baseline were carried over across all follow-up times. X-axis values represent percent difference (with 95% CI) in IC per SD increase for continuous determinants. P values were corrected for the Benjamini-Hochberg false discovery rate. GEE model is adjusted for estimated glomerular filtration rate (eGFR). ALT, alanine aminotransferase; AUC, area under the curve; IC, insulin clearance; T2DM, type 2 diabetes mellitus; WBC, white blood cell count.

**Figure 3:**
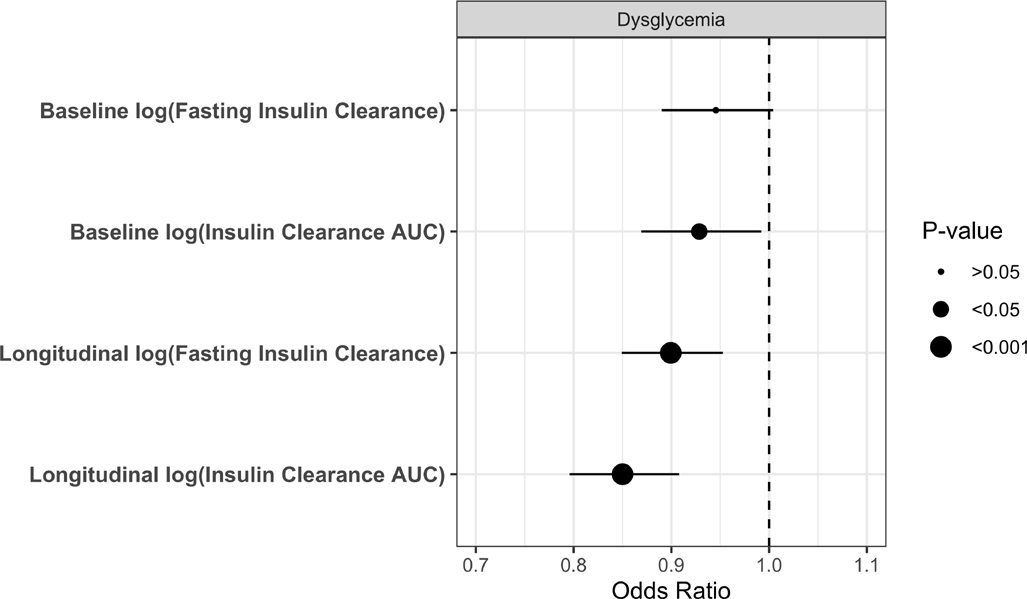
ORs from separate generalized estimating equation (GEE) models showing the association of baseline and longitudinal IC_FASTING_ and IC_AUC_ measures and incident dysglycemia (IGT/IFG/T2D) over the 9-year follow-up in the Prospective Metabolism and Islet Cell Evaluation (PROMISE) cohort. Data adjusted for visit, sex, baseline age, ethnicity, physical activity, smoking status, family history of type 2 diabetes (T2D), white cell count (WCC), alanine aminotransferase (ALT), and estimated glomerular filtration rate (eGFR). AUC, area under the curve; IC, insulin clearance; IFG, impaired fasting glucose; IGT, impaired glucose tolerance.

IC declined over the 9-year follow-up period (20% and 8% for IC_FASTING_ and IC_AUC_, respectively). The main results from multivariate GEE analyses are presented in figures 1–3. Baseline determinants of IC change over the 9-year follow-up period were similar for IC_FASTING_ and IC_AUC_ (figure 1). Non-European ethnicity showed a strong inverse association with IC over time, while controlling for all other determinants in the model (p<0.001). Similarly, baseline measures of WC, WCC, and ALT were negatively associated with IC over time (all p<0.05). Other potential determinants including sex, age, physical activity, smoking status, family history of T2D, and pre-diabetes at baseline did not show a significant relationship with change in IC. There were no significant interaction effects by time in any of the models.

Assessment of longitudinal measures of IC determinants with IC change over time showed similar results (figure 2). Follow-up duration was inversely associated with IC when controlling for all other variables in the model (p<0.001). Likewise, WC, WCC, and ALT were inversely associated with IC measures (p<0.01). Physical activity and age over the 9-year follow-up period showed significant positive associations with both IC_AUC_ and IC_FASTING_ over time, while dysglycemia status was significantly associated with longitudinal decline in IC_AUC_ but not IC_FASTING_. Sex, smoking status, and family history of diabetes were not associated with longitudinal change in IC.

Incident dysglycemia conversion rates were 23%, 15% and 12% at the 3, 6 and 9-year follow-up visits, respectively. Associations of baseline and change in IC with incidence of dysglycemia over the follow-up period were assessed in separate models for each of these measures (figure 3). Fully adjusted models, controlling for visit, sex, baseline age, ethnicity, physical activity, smoking status, family history of T2D, WCC, ALT, and eGFR, showed significant inverse associations between these measures and incidence of dysglycemia, except for IC_FASTING_.

At baseline, participants in the lowest tertiles of insulin sensitivity and beta-cell function had significantly lower IC_FASTING_ and IC_AUC_ compared with those in higher tertiles (all p<0.05). Further, IC in these individuals with poor baseline insulin sensitivity and beta-cell function remained low across follow-up visits at 3, 6, and 9 years (online supplementary figures S2 and S3). In contrast, while participants with higher insulin sensitivity and beta-cell function at baseline showed a significant decline over time in both IC_FASTING_ and IC_AUC_ compared with those in the low and middle tertile groups, these individuals nonetheless had higher IC rates throughout the follow-up period compared with those with the poorest baseline insulin sensitivity and beta-cell function.

## Discussion

Our findings from the PROMISE cohort indicate an overall decline in fasting and OGTT-derived IC measures over the 9-year follow-up period in participants at high risk for T2D development. Furthermore, we identified that components of the metabolic syndrome (including central obesity and markers of inflammation and fatty liver) and non-European ethnicity were independently associated with declines in IC. Finally, lower baseline IC, and declines in IC over time, were related to the incidence of dysglycemia. To our knowledge, this is the first study to assess the longitudinal determinants of change in IC with data from multiple follow-up visits. Our detailed measurements at each follow-up visit allowed for assessment of both fasting and area-under-the-curve IC over time in a high-risk population.

It has been proposed that changes in IC represent a compensatory response to early declines in insulin sensitivity and/or secretion,^9 29^ or, alternatively, are a consequence of upstream metabolic disorders such as ectopic liver fat.^8 15^ A cross-sectional study of 92 healthy, non-diabetic individuals showed a significant decline in IC at each tested glucose infusion rate in insulin-resistant obese participants compared with insulin-sensitive obese subjects; however, there was no significant difference between insulin-sensitive obese and non-obese groups.^30^ In another cross-sectional study of 91 non-diabetic obese subjects, increased insulin secretion and decreased IC rate were concurrently observed in the insulin-resistant group.^31^ Furthermore, declining IC (and not increasing secretion) was the first adaptation to declining insulin sensitivity.^31^ These findings support the notion that declining IC may be a compensatory mechanism to reduced insulin sensitivity. In contrast, however, studies have also shown that upstream risk factors for T2D, such as fatty liver, are independently related to declines in IC.^8 15^ A cross-sectional study of 80 non-diabetic subjects showed that increased liver fat, measured using proton magnetic resonance spectroscopy, was associated with impaired IC.^15^ Similar findings demonstrating declines in IC with increased liver fat have also been observed in diabetic subjects.^8 32^ Evidence to date from human studies, however, has been insufficient to confirm or refute these hypotheses.

Results from the present study suggest the possibility that these pathophysiological phenomena may coexist early in the natural history of T2D. We assessed 9-year changes in IC across baseline tertiles of insulin sensitivity and beta-cell function. Although there was a steeper decline in IC among participants categorized in the highest tertiles of insulin sensitivity and beta-cell function at baseline compared with the other groups, IC was consistently low during follow-up among those with the poorest baseline insulin sensitivity and beta-cell function. Thus, suggesting that IC may be an early adaptation to compromised insulin sensitivity and beta-cell function.

At the same time, our results provide evidence that upstream metabolic disorders contribute to changes in IC. Specifically, we showed that increased ALT concentration, a proxy for ectopic liver fat deposition, was associated with declining IC, suggesting that reduced IC may in part be a consequence of hepatic dysfunction caused by ectopic fat deposition. Similar findings were seen in the Insulin Resistance Atherosclerosis Study (IRAS) Family Study cohort of 1116 participants where ALT declined across increasing tertiles of IC,^2^ and remaining significant after adjustment for age, sex and ethnicity.

To further understand the determinants of IC change over time, we examined the impact of other major risk factors associated with underlying disorders of T2D. We investigated the relationship between IC and central obesity using WC. We identified an inverse longitudinal association between IC and WC, which is consistent with findings from previous studies.^4 6 7 13 33^ Cross-sectional analysis from the IRAS cohort showed that WC declined significantly across increasing tertiles of IC when adjusted for age, sex, ethnicity and center of data collection (p<0.001).^4^ In line with our results, analysis of 800 subjects from the Metabolic Syndrome Berlin Brandenburg study showed that IC was inversely associated with WC (r=−0.28, p<0.001) after adjusting for age and sex.^13^ Furthermore, Erdmann *et al* showed a weight-dependent decrease in IC in 271 subjects stratified by BMI.^33^ Our findings extend the current literature by documenting a longitudinal association of central adiposity (using measurements of WC at multiple time points) with change in IC over time.

Other components of the metabolic syndrome also showed significant longitudinal associations with IC. WCC, a marker of low-grade inflammation, was a significant determinant of longitudinal declines in both IC measures in this study. WCC has previously been associated with obesity and the development of T2D.^34^ Furthermore, we showed that increased physical activity over time was positively associated with increased IC, a finding consistent with current literature.^16 35 36^ A weight loss experiment of 15 obese children and adolescents tested the effects on IC at various levels of strenuous exercise over a 10-week period.^36^ Compared with baseline, all subjects lost weight and IC improved significantly. Interestingly, improvements in IC have been observed immediately after 2 hours of strenuous exercise in healthy and diabetic men.^35^ Similar findings of acute physical activity and improved IC have been observed in mice.^16^ The physiological pathway through which physical activity improves IC remains unclear; however, it has been hypothesized that the physical activity increases the expression of IDE.

In addition to determinants associated with inflammation and the development of metabolic syndrome, ethnicity may be an important factor in IC response. Previous research has shown that IC declines more steeply in those of non-Caucasian ethnicity.^4 37^ A cross-sectional study showed that African-American children experienced a greater IC decline than American White children (p<0.001).^37^ Similarly, the multiethnic IRAS cohort reported a significantly larger proportion of African-American and Hispanic subjects versus non-Hispanic Whites in the lowest tertile of IC compared with the highest tertile.^4^ Although our population was over 70% European ethnic origin, we observed a clear distinction between non-European versus European at the extreme tertiles of IC at baseline and relationship was significant in our longitudinal analysis.

In this study, we saw an inverse association of IC with incident dysglycemia. The relationship of insulin sensitivity and beta-cell function with declining glucose tolerance has been widely studied,^38 39^ but less information is available regarding the role of IC. A previous experiment examined glucose tolerance in elderly subjects and showed elevated glucose levels in elderly compared with young participants. These findings were associated with defects in insulin sensitivity, beta-cell function, and IC.^40^ Specifically, compared with young participants, the elderly subjects had lower total body IC suggesting a possible compensatory response to glucose intolerance. Furthermore, consistent with our findings, hepatic IC increased in the elderly subjects compared with younger subjects, suggesting that total body and hepatic IC are regulated differently.

One of the main strengths of this study is that PROMISE is a well-characterized multiethnic longitudinal cohort of subjects at risk for T2D development. Detailed assessments at baseline and multiple follow-ups allowed for the consideration of repeated measurements of IC, insulin sensitivity, beta-cell function, and covariates. In addition, the GEE statistical model used in our longitudinal analysis helped retain the maximum number of subjects.

There are, however, a few limitations to consider. Our assessment of IC, insulin secretion, and beta-cell function was not captured using gold standard procedures; the invasiveness and cost of those approaches are not amenable for large cohorts. Instead, validated proxy measures were calculated using insulin, glucose, and C peptide values from fasting and OGTT administered at each visit. Our method of calculating IC has been used in other studies^13 21 22 41^ and IC_AUC_ has been shown to be reflective of IC measured using hyperinsulinemic-euglycemic clamps (r=0.74, p<0.001).^22^ Furthermore, indices used to estimate insulin sensitivity and IC were derived in part from the same core variables (specifically, insulin concentrations during the OGTT) and thus we were not able to evaluate their association in the same GEE models due to collinearity. However, we evaluated the relationship of well-established proxies of insulin sensitivity, such as WC, with changes in IC in these models. Also, other potentially important variables may affect changes in IC, which we were unable to account for. Genetics may be an important player in IC. A study of 513 Mexican-Americans demonstrated that changes in IC may be a heritable trait with the identification of chromosomes 15 and 20 related to IC.^42^ Additionally, with this being an observational cohort, we are not able to confirm causality due to the potential for residual confounding. Furthermore, the PROMISE cohort does not include participants who were completely free of risk factors for T2D at baseline. Lastly, the generalizability of our findings is limited to individuals with similar demographic characteristics as this population.

In conclusion, our findings provide evidence for the role of core components of the metabolic syndrome, specifically fatty liver and subclinical inflammation, as factors determining longitudinal declines in IC. In addition, we saw that IC was associated with declining insulin sensitivity and beta-cell function, and, importantly, the trajectory of decline depended on the status of these variables at baseline. Therefore, our study highlights the complexity in factors that impact the decline in IC. Changes in IC may be due to an interplay of both upstream metabolic abnormalities and compensatory responses to early metabolic disorders associated with T2D. Our current results extend existing literature regarding IC by using a longitudinal design with repeated measures at four time points. Additional longer term studies are needed to further expand our understanding of the role of IC in the natural history of T2D.
