# Supplementary material for: Determinants of longitudinal change in insulin clearance: the Prospective Metabolism and Islet Cell Evaluation cohort
Source: BMJ Open Diabetes Res Care. 2019 Nov 24;7(1):e000825. doi: 10.1136/bmjdrc-2019-000825 (PMC6887510; doi:10.1136/bmjdrc-2019-000825)
Supplement: Supplementary data [file bmjdrc-2019-000825supp001.pdf]

Table S1. Formulae used to calculate insulin sensitivity and beta-cell function.

Measure	Formula
**HOMA2-%S**	Determined using the University of Oxford Diabetes Trials Unit online excel spreadsheet.[1,2]
**ISI[3]**	$$ISI = \frac{10000}{\sqrt{(Glucose_{0min} \times Insulin_{0min}) \times (Glucose_{mean} \times Insulin_{mean})}}$$
**ISSI-2[4]**	$$ISSI - 2 = (\frac{Insulin\ AUC}{Glucose\ AUC}) \times ISI$$
**IGI/HOMA-IR[5]**	$$IGI/HOMA - IR = \frac{\frac{Insulin_{30min} - Insulin_{0min}}{Glucose_{30min} - Glucose_{0min}}}{\frac{Glucose_{0min} - Insulin_{0min}}{22.5}}$$

[1] Levy JCM, D. R. Hermans, M. P. Correct homeostasis model assessment (HOMA) evaluation uses the computer program. *Diabetes Care.* 1998;21(12):2191-2.

[2] https://www.dtu.ox.ac.uk/homacalculator/download.php

[3] Matsuda MD, R. A. Insulin sensitivity indices obtained from oral glucose tolerance testing: Comparison with the euglycemic insulin clamp. *Diabetes Care.* 1999;22(9):1462 – 70.

[4] Retnakaran RS, S. Hanley, A. J. Vuksan, V. Hamilton, J. K. Zinman, B. Hyperbolic relationship between insulin secretion and sensitivity on oral glucose tolerance test. *Obesity (Silver Spring).* 2008;16(8):1901-7.

[5] Wareham NJP, D. I. Byrne, C. D. Hales, C. N. The 30 minute insulin incremental response in an oral glucose tolerance test as a measure of insulin secretion. *Diabet Med.* 1995;12(10):931.

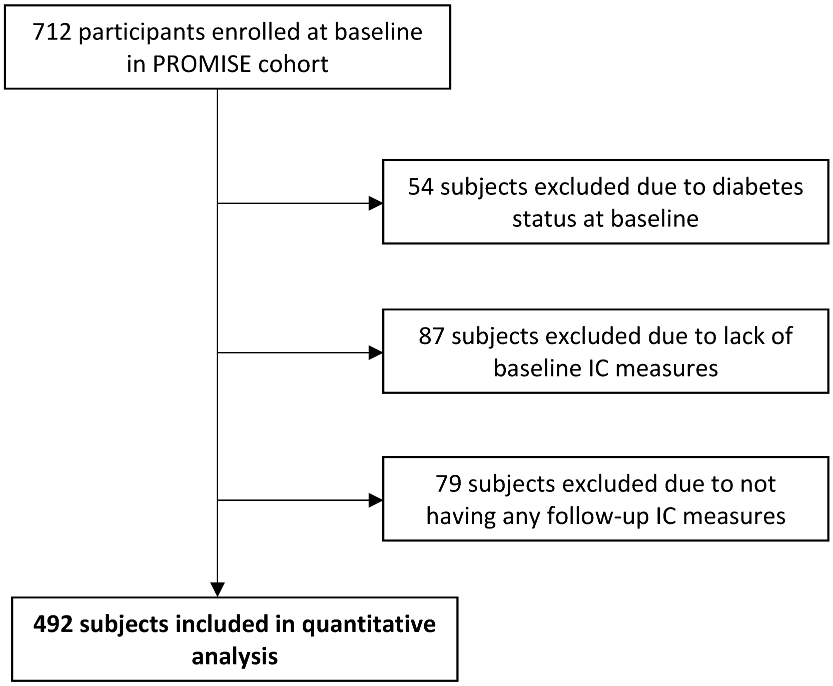

Figure S1. Consort diagram outlining the summary of subject exclusion and inclusion. Of the 712 PROMISE participants at baseline, 220 were excluded based on prespecified criteria. The remaining 492 subjects were analyzed.

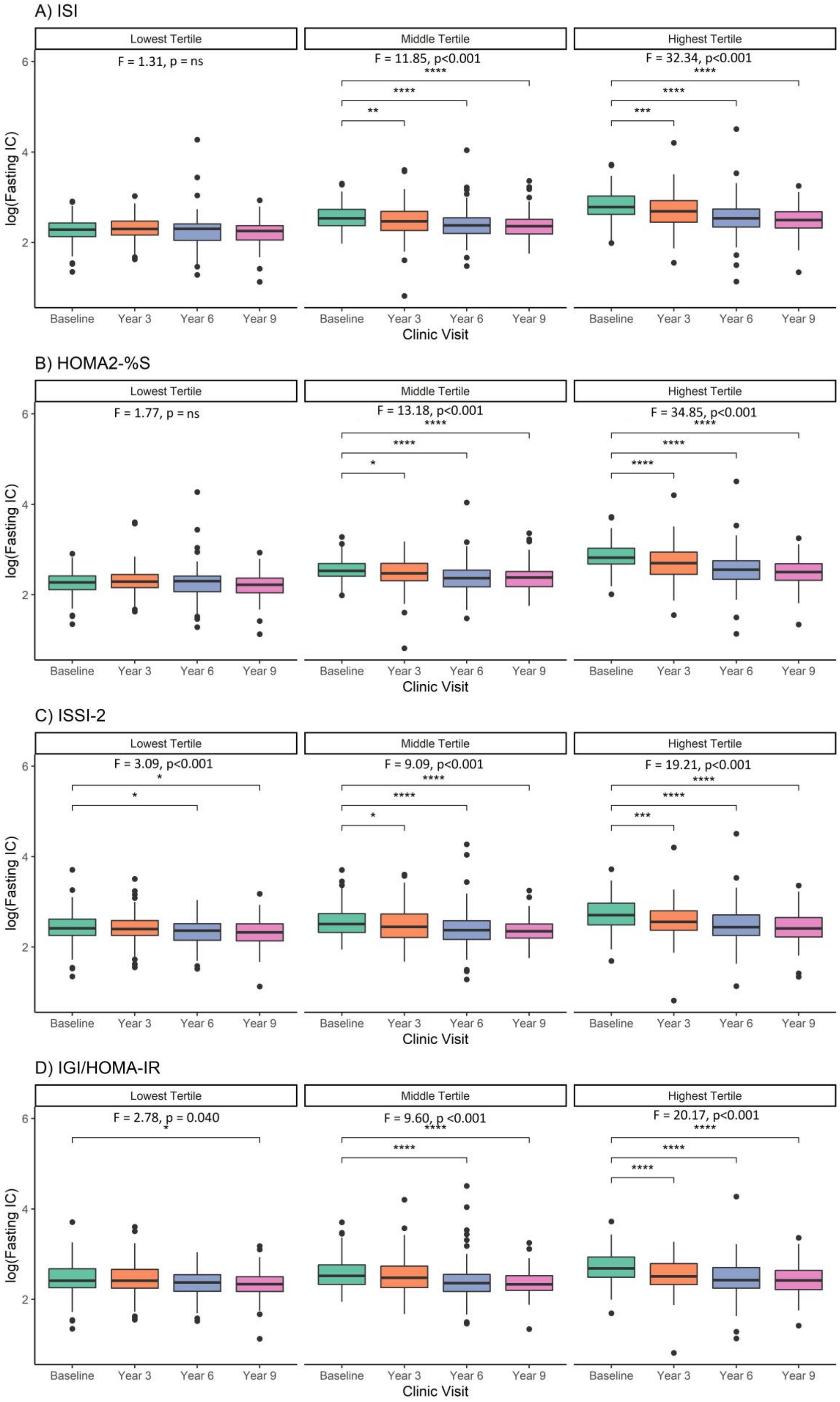
A) ISI
Lowest Tertile
Middle Tertile
Highest Tertile
F = 1.31, p = ns
F = 11.85, p<0.001
F = 32.34, p<0.001
log(Fasting IC)
Baseline
Year 3
Year 6
Year 9
Clinic Visit
B) HOMA2-%S
F = 1.77, p = ns
F = 13.18, p<0.001
F = 34.85, p<0.001
C) ISSI-2
F = 3.09, p<0.001
F = 9.09, p<0.001
F = 19.21, p<0.001
D) IGI/HOMA-IR
F = 2.78, p = 0.040
F = 9.60, p <0.001
F = 20.17, p<0.001

Figure S2. Longitudinal trends in $IC_{FASTING}$ according to tertiles of baseline insulin sensitivity and beta-cell function. A: ISI; B: HOMA2-%S; C: ISSI-2; D: IGI/HOMA-IR. F-statistics and p-values shown are from ANOVA tests for each tertile. Symbols within the figure indicate p-values from pairwise tests: * p<0.05, ** p<0.01, ***p<0.001, **** p<0.001.

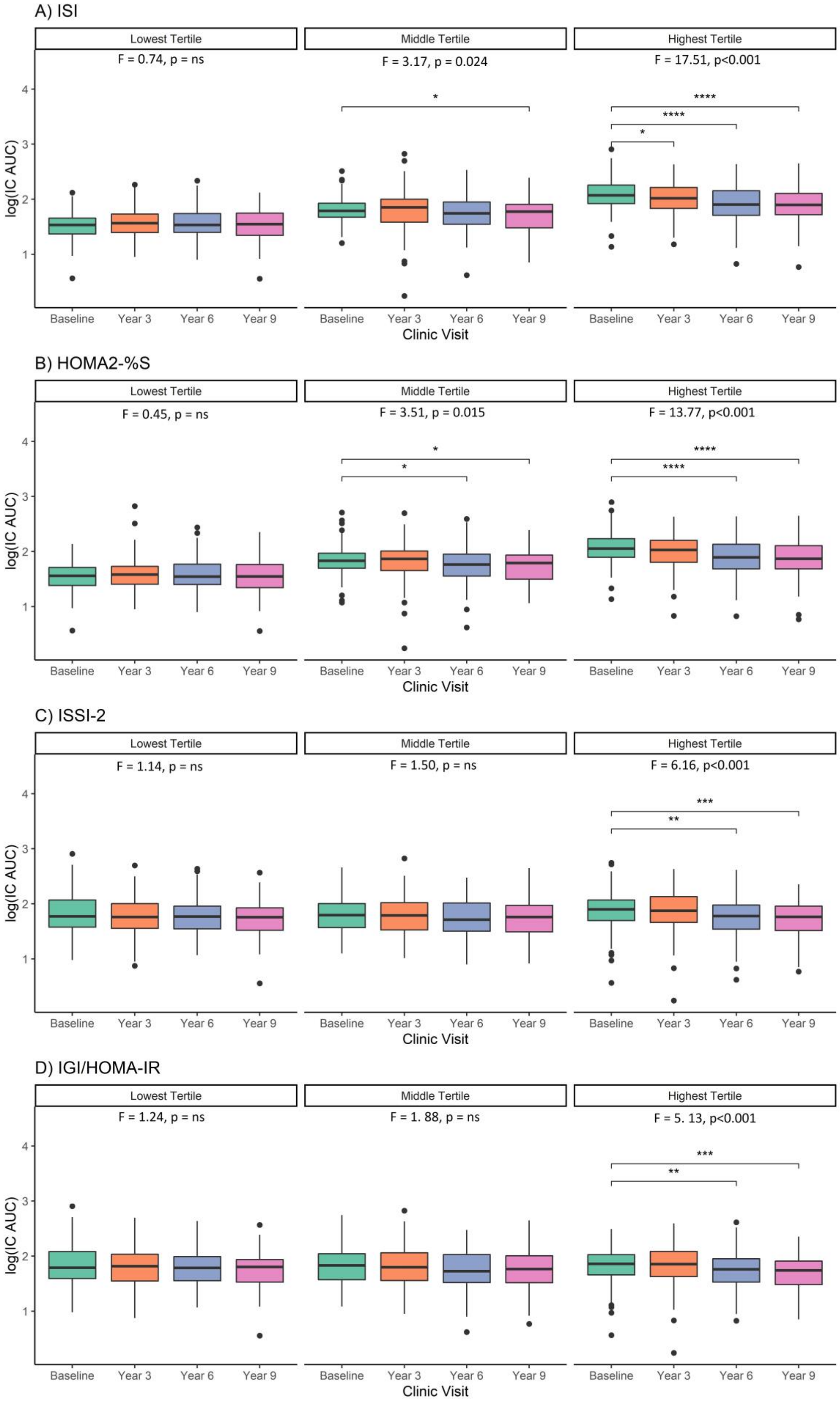
A) ISI
Lowest Tertile
F = 0.74, p = ns
Middle Tertile
F = 3.17, p = 0.024
Highest Tertile
F = 17.51, p<0.001
log(IC AUC)
Baseline
Year 3
Year 6
Year 9
Clinic Visit
B) HOMA2-%S
Lowest Tertile
F = 0.45, p = ns
Middle Tertile
F = 3.51, p = 0.015
Highest Tertile
F = 13.77, p<0.001
C) ISSI-2
Lowest Tertile
F = 1.14, p = ns
Middle Tertile
F = 1.50, p = ns
Highest Tertile
F = 6.16, p<0.001
D) IGI/HOMA-IR
Lowest Tertile
F = 1.24, p = ns
Middle Tertile
F = 1. 88, p = ns
Highest Tertile
F = 5. 13, p<0.001

Figure S3. Longitudinal trends in IC_{AUC} according to tertiles of baseline insulin sensitivity and beta-cell function. A: ISI; B: HOMA2-%S; C: ISSI-2; D: IGI/HOMA-IR. F-statistics and p-values shown are from ANOVA tests for each tertile. Symbols within the figure indicate p-values from pairwise tests: * p<0.05, ** p<0.01, ***p<0.001, **** p<0.001.
